# Age-Related Changes of the Human Crystalline Lens on High-Spatial Resolution Three-Dimensional T1-Weighted Brain Magnetic Resonance Images In Vivo

**DOI:** 10.1167/iovs.61.14.7

**Published:** 2020-12-03

**Authors:** Felix Streckenbach, Oliver Stachs, Sönke Langner, Rudolf F. Guthoff, Felix G. Meinel, Marc-André Weber, Thomas Stahnke, Ebba Beller

**Affiliations:** 1 Institute of Diagnostic and Interventional Radiology, Pediatric Radiology and Neuroradiology, Rostock University Medical Center, Rostock, Germany; 2 Department of Ophthalmology, Rostock University Medical Center, Rostock, Germany; 3 Centre for Transdisciplinary Neurosciences Rostock, University of Rostock, Rostock, Germany

**Keywords:** age-related changes, lens development, magnetic resonance imaging

## Abstract

**Purpose:**

To reveal age-related changes of the human crystalline lens by using high-spatial resolution T1-weighted brain magnetic resonance imaging of patients under general anesthesia.

**Methods:**

We retrospectively identified 47 children (2–17 years) and 30 adults (18–70 years) without diabetes or eye disease, who required brain magnetic resonance imaging examinations under general anesthesia between 2012 and 2019. Normalized signal intensity of the crystalline lens and vitreous body, as well as equatorial diameter and axial thickness of the lens were assessed by using a three-dimensional T1-weighted magnetization prepared rapid acquisition gradient echo sequence of the brain with 0.9-mm spatial resolution. Patient dossiers were reviewed to record indication for magnetic resonance imaging examination and hypertension.

**Results:**

Advancing age was significantly correlated with increasing equatorial diameter of the infantile lens (r = 0. 74; 95% confidence interval, 0.58–0.85; *P* < .0001) and increasing crystalline lens signal intensity of the adult lens (r = 0.38; 95% confidence interval, 0.02–0.65; *P* = .0382), which remained significant after accounting for potential confounding variables. There was no significant correlation between age and axial thickness or vitreous body signal intensity in the children and adult cohort.

**Conclusions:**

The present study demonstrated that advancing age was significantly correlated with an increasing equatorial diameter of the infantile lens and with increasing crystalline lens signal intensity of the adult lens. These normative data can contribute to our understanding of age-related changes in eye health and function, especially in regard to the emmetropization process and should also be taken into account when investigating lens pathologies.

The human crystalline lens is the ocular component whose changes are most pronounced with age.^1^ It would, therefore, seem that the changes of the lens, which determine its optical characteristics, play an important part in emmetropization (maintaining refraction in the normal range through compensatory mechanisms).^2^ Moreover, the crystalline lens continues to grow beyond the time that initial emmetropia is obtained.^3^ Because the process of emmetropization and the maintenance of emmetropia are most probably affected by changes in lens size, shape, and mass, it is important to carefully characterize the growth pattern.^4^ Additionally, not only growth but also constant remodeling of its components and structure make the lens an interesting tissue for the study of aging and aging-associated pathologies.^5^

Compared with current clinical technologies, such as optical low-coherence reflectometry, optical coherence tomography, partial optical coherence interferometry or ultrasonic A-scan, magnetic resonance imaging (MRI) offers a unique advantage to investigate the human crystalline lens geometry noninvasively and in vivo without affecting the measured parameters.^5^^–^^7^ MRI examinations under sedation, especially of pediatric patients, have the advantage that limited attention span, and thus a limited ability to focus on a target and remain still, need not to be taken into consideration.

Therefore, the aim of this study was to reveal normative data and age-related changes of the human crystalline lens in children and adults by using a high-spatial resolution three-dimensional (3D) T1-weighted magnetization prepared rapid acquisition gradient echo (MPRAGE) sequence of patients under general anesthesia who required brain MRI examinations.

## Methods

### Study Design and Ethical Approval

The study was designed as a retrospective, single-center cohort study. Inclusion criteria included patients (1) aged between 2 and 70 years who (2) required brain MRI examinations under general anesthesia at our institution between January 2012 and October 2019, (3) which included a native 3D T1-weighted MPRAGE sequence with 0.9-mm spatial resolution. Exclusion criteria included patients with (1) a history of diabetes and (2) known eye disease as well as datasets with (3) motion artifacts. Patients younger than 2 years old were excluded in an attempt to attenuate the effect of preterm births on lens parameters.^8^ The study population was then divided into a children cohort (2–17 years) and an adult cohort (18–70 years).

The study protocol was approved by the responsible institutional review board with a waiver of informed consent and was conducted in compliance with the Declaration of Helsinki in its current form.

### Patient Selection

We identified eligible patients through a retrospective search of our radiology information system (Centricity 5.0, GE Healthcare, Barrington, IL). All consecutive patients meeting all the inclusion criteria and none of the exclusion criteria were included in the analysis. A review of patient dossiers was performed to record the indication for MRI examination, a history of diabetes, eye disease, or hypertension at the time of the MRI examination.

### MRI Examination Protocol

**Figure 1.**
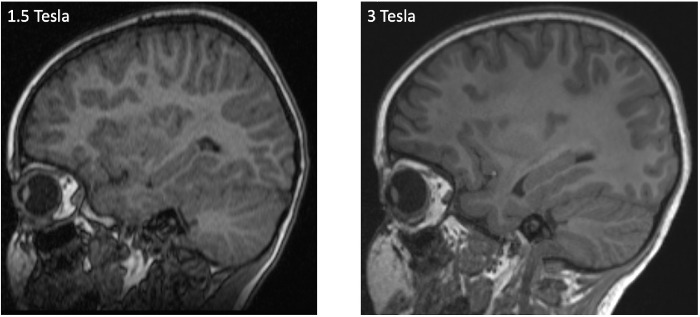
Representative example of 3D MPRAGE brain images of a 5-year old female patient at 1.5 Tesla and a 4-year-old female patient at 3 Tesla.

All brain MR examinations were performed on 3 Tesla systems (Magnetom Verio and Magnetom Skyra fit, Siemens Healthineers, Erlangen, Germany) or on 1.5 Tesla systems (Magnetom Avanto and Magnetom Avanto Fit, Siemens, Healthineers) using a 12- or 32-channel head coil for signal detection (Fig. 1). The 3D T1-weighted MPRAGE sequence with 0.9-mm spatial resolution were part of the MRI protocol included in our study. For a detailed description of the acquisition parameters, please see Supplementary Table S1. All datasets of the examination were archived in our PACS (IMPAX 6.5.3, Agfa HealthCare, Bonn, Germany). All brain MRI examinations were performed while under general anesthesia, which included a continuous propofol infusion.

### Image Analysis

**Figure 2.**
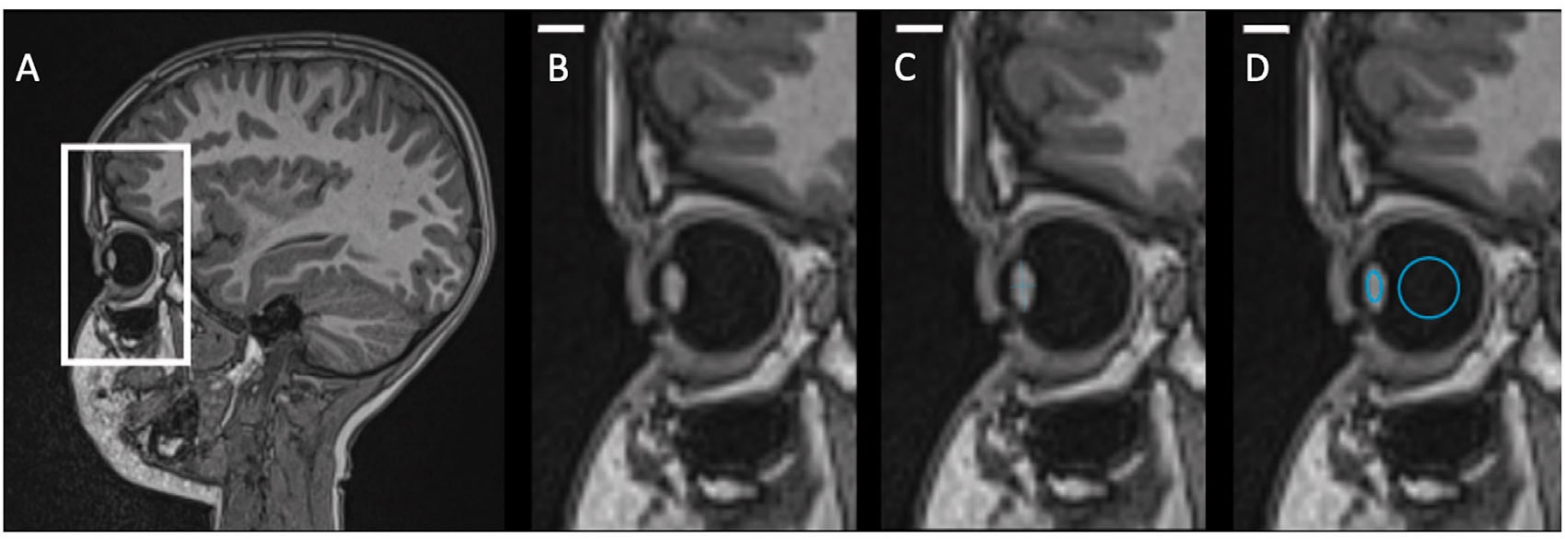
Representative example of an unenhanced T1-weighted 3D MPRAGE brain image in the sagittal plane of a 6-year-old male patient (**A**). The right eye is magnified (bar ≙ 7 mm) (**B**). Lens measurements were performed (**C**) as well as the region of interest measurement of the mean signal intensity were drawn on the lens and vitreous body (**D**).

Image quality was assessed visually and independently by two radiologists (one fellow, one board-certified attending neuroradiologist). First, both examiners distinguished between sufficient or insufficient image quality for analysis. For image evaluation, multiplanar reformats of unenhanced 3D MPRAGE images perpendicular to the longest and shortest axes of the right lens were reconstructed also independently by two radiologists using a 3D module within our PACS. The following length measurements of the right eye were then performed manually: equatorial diameter (6–12 o'clock axis of the lens) and axial lens thickness (from anterior to posterior pole) of the crystalline lens with axial thickness being perpendicular to equatorial diameter. Additionally, the mean T1 signal intensity of the crystalline lens, the vitreous body and cerebrospinal fluid in the fourth ventricle were examined independently by two radiologists using a standardized elliptic region of interest with the same size of 8 mm^2^ for lens and cerebrospinal fluid and a circular region of interest with the same size of 50 mm^2^ for the vitreous body for all patients. ROIs were carefully placed in the center of the lens, vitreous body, and the fourth ventricle in the sagittal plane on unenhanced 3D MPRAGE images (Fig. 2). The mean T1 signal intensity ratio of the crystalline lens and vitreous body was calculated by dividing the mean signal intensity of the crystalline lens or vitreous body by the mean signal intensity of the cerebrospinal fluid.

### Statistical Analysis

Statistical analysis was performed with GraphPad Prism 5 and SPSS statistics (version 20). Cohen's kappa coefficient (κ) was used to study the agreement between both readers regarding image quality and intraclass correlation (ICC) in a two-way random, consistency, average measure approach to assess the inter-rater reliability for measurements of the lens and vitreous body. Agreement was considered excellent, substantial, moderate, or weak when the kappa or ICC values were above 0.75, between 0.75 and 0.60, between 0.60 and 0.40, or less than 0.40, respectively.^9^^,^^10^ Pearson's correlation coefficient (r) was determined to assess the relationship of age with equatorial diameter, axial thickness, and normalized signal intensity of the crystalline lens and vitreous body.^11^ Linear regression analyses were used to examine whether age-related changes of the equatorial diameter in the children cohort and of the normalized T1 signal intensity of the crystalline lens in the adult cohort remained significant after accounting for potential confounding variables. *P* values of less than .05 were regarded as statistically significant.

## Results

### Patient Characteristics

**Figure 3.**
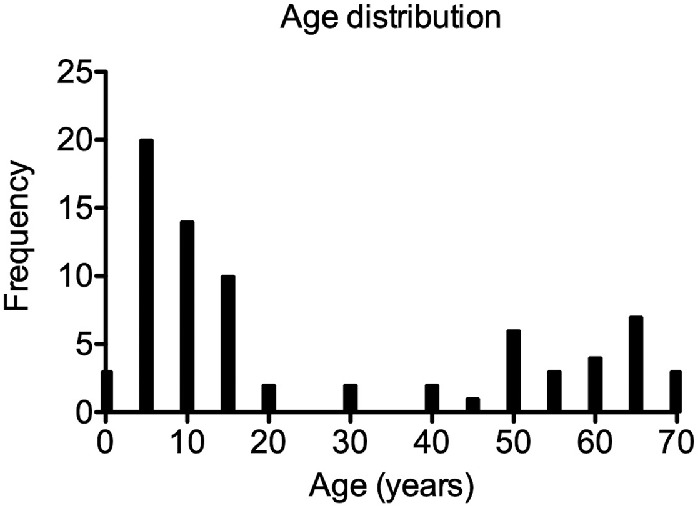
Histogram of the age distribution of the study population (2–70 years, *n* = 77).

There were 428 patients identified, who required brain MRI examinations under general anesthesia at our institution between January 2012 and October 2019. Patients aged less than two years or more than 70 years (*n* = 152), with no native high-spatial resolution 3D T1-weighted MPRAGE sequence included in the MRI protocol (*n* = 167) or reduced image quality owing to motion artifacts (*n* = 27), as well as a history of diabetes (*n* = 5) or eye disease (*n* = 0) were excluded. The final study population consisted of a total of 77 patients and was subdivided into a children cohort (2–17 years) with 47 subjects and an adult cohort (18–70 years) with 30 subjects. The most common indication for obtaining brain MRI examination under general anesthesia in the children cohort included seizure (*n* = 16), inflammatory disease, mostly meningitis or encephalitis (*n* = 12), as well as brain tumor (*n* = 8). The most common MRI indications in the adult cohort were brain tumor (*n* = 22), seizure (*n* = 3), and intracranial hemorrhage (*n* = 3). Hypertension was only found in the adult group with 27% (*n* = 8). For a detailed description of the study population, please see the histogram of age distribution (Fig. 3) and Table 1.

**Table 1.** Patient Characteristics

	Total (2–70 Years)	Children Cohort (2–17 Years)	Adult Cohort (18–70 Years)
No.	77	47	30
Age, years, median (IQR)	14 (6–51)	8 (4–12)	55 (47–65)
Female	35 (45)	24 (51)	11 (37)
Hypertension	8 (10)	0 (0)	8 (27)
Most common MRI indications			
Brain tumor	30 (39)	8 (17)	22 (73)
Seizure	19 (25)	16 (34)	3 (10)
Inflammatory disease	14 (18)	12 (26)	2 (7)
Intracranial hemorrhage	8 (10)	5 (11)	3 (10)

Values are number (%) unless otherwise indicated.

### 

#### Inter-Rater Reliability

**Figure 4.**
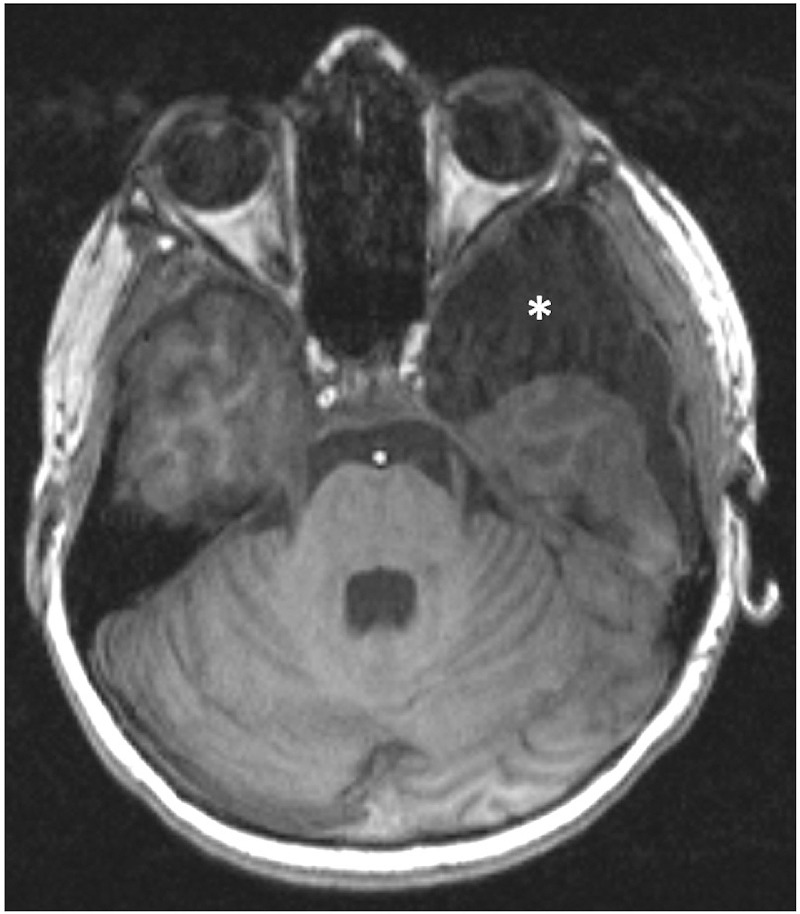
Representative example of an unenhanced 3D MPRAGE brain image with motion artifacts of the lens and vitreous body owing to eye movements, which was rated as insufficient by both readers. MRI also demonstrated a left temporal lobe arachnoid cyst (*).

The agreement between both readers, whether the image quality was sufficient or insufficient for analysis, was very strong (κ  =  0.93) (Fig. 4). Moreover, inter-rater reliability for the equatorial diameter and axial thickness of the crystalline lens was substantial with an ICC of 0.724 (95% confidence interval [CI], 0.566–0.825) and 0.642 (95% CI, 0.437–0.773), respectively. The ICC was 0.849 (95% CI, 0.762–0.904) of the normalized T1 signal intensity of the crystalline lens and 0.968 (95% CI, 0.950–0.980) of the normalized T1 signal intensity of the vitreous body, each indicating an excellent inter-rater reliability.

#### Effect of Age on Lens Size and Normalized T1 Signal Intensity of the Lens and Vitreous Body

**Figure 5.**
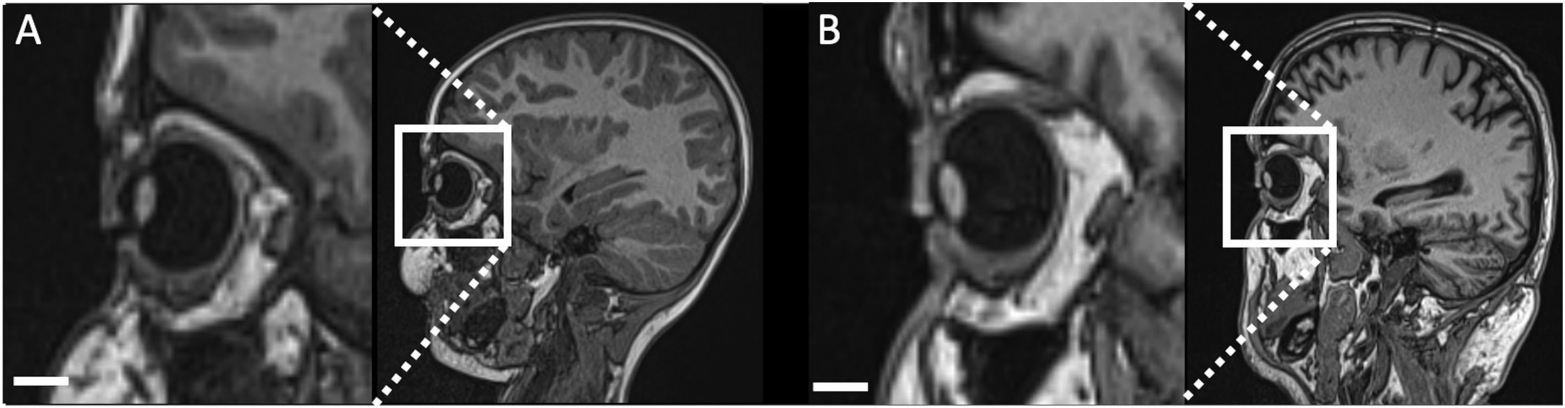
Representative examples of unenhanced T1-weighted 3D MPRAGE brain images in the sagittal plane of a 4-year-old female patient (**A**) and a 65-year-old male patient without hypertension (**B**) with the right eye magnified in the left panel (bar ≙ 7 mm).

**Figure 6.**
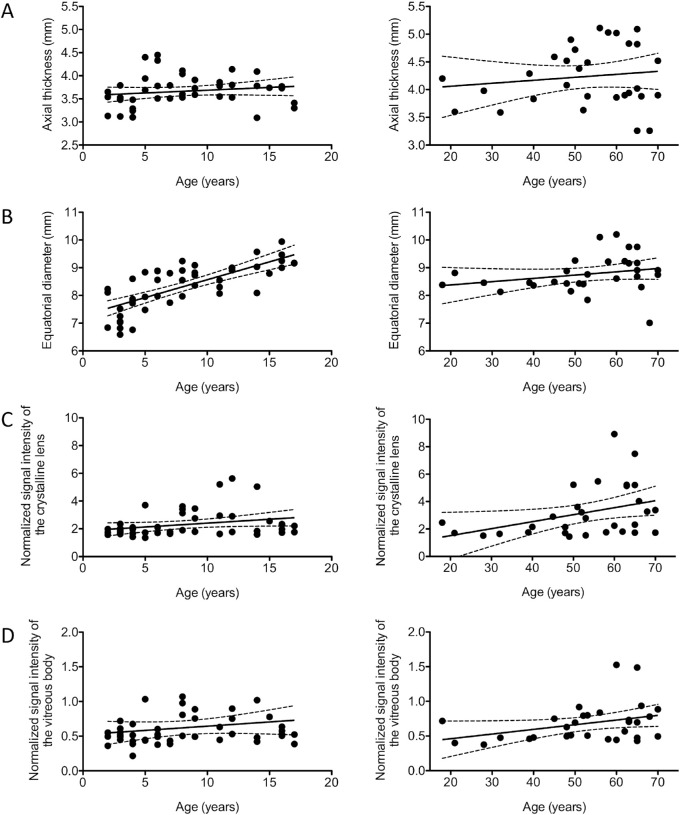
Scatterplots of age against (**A**) axial thickness, (**B**) equatorial diameter, (**C**) normalized signal intensity of the crystalline lens, and (**D**) normalized signal intensity of the vitreous body in the children cohort (*left row*) and adult cohort (*right row*). The regression is shown by the *solid lines* and the 95% confidence interval by the *dashed lines*. Only the equatorial diameter increased significantly with age in the children cohort (r = 0.74; 95% confidence interval, 0.58–0.85; *P* < .0001) and normalized signal intensity of the crystalline lens in the adult cohort (r = 0.38; 95% confidence interval, 0.02–0.65; *P* = .0382).

Advancing age showed a significant positive correlation with axial thickness (r = 0.55; 95% CI, 0.38–0.70; *P* < .0001), equatorial diameter (r = 0.38; 95% CI, 0.17–0.56; *P* = .0006) and normalized signal intensity of the crystalline lens (r = 0.40; 95% CI, 0.20–0.57; *P* = .0003) (Table 1 and Fig. 5). However, subdividing into children (2–17 years) and adults (18–70 years) revealed that a positive correlation between age and equatorial diameter was only significant in the children cohort (r = 0. 74; 95% CI, 0.58–0.85; *P* < .0001) and between age and normalized T1 signal intensity of the crystalline lens was only significant in the adult cohort (r = 0.38; 95% CI, 0.02–0.65; *P* = .0382) (Fig. 6 and Table 1). A significant correlation was neither found between age and axial thickness nor between age and normalized T1 signal intensity of the vitreous body in both groups (Table 1).

#### Linear Regression Analysis

To control for the possibility that the age-related changes of the lens in the children and adult cohort was due to other confounding variables, a linear regression analysis was performed by using the equatorial diameter as a criterion in the children cohort and the normalized T1 signal intensity of the crystalline lens in the adult cohort. The results are summarized in Table 2^3^. Even when controlling for gender and MRI indication in children and also hypertension in adults, the effect of age on the equatorial diameter in the children cohort and on the normalized T1 signal intensity of the crystalline lens in the adult cohort remained significant (*P* < .0001 and .036, respectively). The control variables did not have a significant influence on the equatorial diameter in the children group and on the normalized T1 signal intensity of the crystalline lens in the adult group (Table 3).

**Table 2.** Measurements of the Lens and the Vitreous Body

		Total	Children Cohort	Adult Cohort
AT	r (95% CI)	0.55 (0.38 to 0.70)	0.17 (−0.12 to 0.44)	0.14 (−0.23 to 0.48)
	*P* value	<.0001	.2508	.4627
	Slope (±SE)	0.012 (±0.002)	0.012 (±0.010)	0.005 (±0.007)
	Intercept (±SE)	3.586 (±0.072)	3.568 (±0.100)	3.954 (±0.394)
ED	r (95% CI)	0.38 (0.17 to 0.56)	0.74 (0.58 to 0.85)	0.25 (−0.12 to 0.56)
	*P* value	.0006	<.0001	.1797
	Slope (±SE)	0.013 (±0.004)	0.129 (±0.017)	0.012 (±0.009)
	Intercept (±SE)	8.185 (±0.123)	7.279 (±0.164)	8.143 (±0.468)
CL-SI	r (95% CI)	0.40 (0.20 to 0.57)	0.27 (−0.02 to 0.52)	0.38 (0.02 to 0.65)
	*P* value	.0003	.0665	.0382
	Slope (±SE)	0.025 (±0.006)	0.056 (±0.030)	0.051 (±0.023)
	Intercept (±SE)	2.021 (±0.226)	1.850 (±0.283)	0.497 (±1.277)
VB-SI	r (95% CI)	0.17 (−0.05 to 0.38)	0.17 (−0.12 to 0.44)	0.34 (−0.02 to 0.62)
	*P* value	.1339	.2564	.0654
	Slope (±SE)	0.002 (±0.002)	0.012 (±0.011)	0.007 (±0.004)
	Intercept (±SE)	0.587 (±0.053)	0.523 (±0.100)	0.326 (±0.192)

AT, axial thickness; CI, confidence interval; CL-SI, normalized T1-signal intensity of the crystalline lens; ED, equatorial diameter; r, Pearson's correlation coefficient; SE, standard error; VB-SI, normalized T1-signal intensity of the vitreous body.

**Table 3.** Results of Linear Regression Analyses

Parameter	Regression Coefficient	95% CI	Standardized Regression Coefficient	*P* Value
Children cohort, ED as criterion				
Age	0.126	0.088 to 0.163	0.743	<.0001
Gender	−0.131	−0.475 to 0.213	−0.081	.446
MRI indication	0.018	−0.062 to 0.099	0.048	.649
Adult cohort, CL-SI as criterion				
Age	0.012	0.004 to 0.101	0.391	.036
Gender	1.175	−0.229 to 2.579	0.304	.097
MRI indication	−0.038	−0.467 to 0.391	−0.032	.857
Hypertension	0.507	−1.039 to 2.054	0.121	.505

CI, confidence interval; CL-SI, normalized T1-signal intensity of the crystalline lens; ED, equatorial diameter.

## Discussion

Our data showed that advancing age was significantly correlated with increasing equatorial diameter of the infantile lens and with an increasing normalized T1 signal intensity of the adult lens. The effect of age on the crystalline lens, regarding equatorial diameter in the children cohort and normalized T1 signal intensity in the adult cohort, remained significant after accounting for potential confounding variables.

Despite changes in ocular biometric components with age, notably the lens, the visual system manages to achieve and maintain emmetropization.^4^ However, if imbalance among these components occurs, it is the main cause for refractive errors.^12^ Therefore, an accurate description of age-related changes regarding parameters of the lens might be helpful for a better understanding of the emmetropization process. To our knowledge, this study is the first to assess lens size and signal intensity on MR images that were acquired under general anesthesia with propofol. Under propofol anesthesia, the eye is most likely in an unaccommodated state analogous to the unaccommodated noncycloplegic refractive state in conscious human subjects.^13^^,^^14^ Moreover, eye movement is generally decreased under propofol anesthesia.^15^ However, we found a higher amount of MR images with motion artifacts in our study compared with other studies, for example, using a clued blinking protocol.^16^ Nevertheless, propofol anesthesia is found to reduce ocular microtremor, a constant, physiologic, high-frequency tremor of the eyes linked to neural activity in the brainstem^17^ that, although with low amplitude, may also contribute to motion artifacts seen on MRI.^15^ Furthermore, MRI examinations under sedation allow unique data collection from children across a wide range of ages. This study would be very complicated in conscious children (especially very young children) because of their limited attention span and urge to move around. Additionally, ex vivo or more direct, invasive methods of lenticular measurements used to date suffer from the disadvantage that they may affect the measured parameters.^1^

Previous studies had inconclusive results regarding age-related changes of axial thickness of the infant lens. Similar to our results, no significant changes in lens thickness was found in a study of children aged 1 month to 6 years in Japan^18^ and aged 6 to 16 years in Tibet.^19^ However, an age-related decrease in the lens thickness was observed in Iranian and American children between the ages of 6 and 18 years^12^^,^^20^ and between 6 and 14 years.^21^ Interestingly, a study from Taiwan suggested lens thinning between the ages of 7 and 11 years to compensate for increased axial length of normal eye growth and subsequent increase of lens thickness correlated with years.^22^ Inconsistencies between the studies might be due to differences of the study cohort regarding the age span, measurement techniques (MRI vs. ultrasound vs. optical low coherence reflectometry), and the race of the studied population, as well as the prevalence of refractive errors.^20^

Our findings revealed only a small increase in axial thickness of 0.005 mm/year in the adult cohort, which was not significant. However, most studies reported a significant increase of lens thickness with age in adults at a rate between 0.018 and 0.024 mm/year in the accommodated state.^23^^–^^26^ This surprising finding of our study may be due to an uneven distribution of the adult data across age groups, particularly the paucity of prepresbyopic adults. It may also be due to the small number of subjects, lower resolution compared with ocular MRI, or instability of accommodative state during anesthesia.

In our study, any age-related change in the adult lens equator was not statistically significant, perhaps owing to the limitations discussed elsewhere in this article. Prior MRI studies of conscious adults find the lens equator either constant or increasing with age, depending on the accommodative state.^23^^,^^26^ In contrast, advancing age showed a significant positive correlation with equatorial diameter in the children cohort. These results are similar to the results by Ishii et al,.^18^ who also evaluated the lens size of children who underwent brain MRI under sedation. However, an exact comparison cannot be made because their study cohort included children with the age of 1 month to 6 years, greater slice thickness between 1.2 and 2.4 mm, and sedation was performed with triclofos sodium syrup.^18^ However, both studies support the idea that the maintenance of emmetropia in the growing eye occurs through stretching of the distensible crystalline lens.^18^^,^^27^

Patients with traumatic or diabetic cataracts and osmotic cataract animal models display T1 and T2 relaxation times changes of the lens observable on MRI.^28^^,^^29^ These changes include decreased signal on T1-weighted sequences and increased signal on T2-weighted sequences, which may be attributed to increased hydration of the lens.^28^ In our study, normalized signal intensity of the lens on T1-weighted images was significantly increased with age in the adult cohort but not in the children cohort. These results should, however, be interpreted with caution, because T1 signal intensity not only depends on the tissue itself, but is also an interplay of multiple acquisition parameters, including the shot interval between inversion pulses, inversion time, and flip angle.^30^ Therefore, it can only be speculated that these findings might result from an opposite effect owing to decreased diffusion of water from the outside to the inside of the lens^31^ and a decreased percentage of bound water in all layers of the crystalline lens, which occur later in life.^5^ Yet, several studies showed that the total water content of the lens did not alter with age.^32^^,^^33^ Age-related changes in lens composition, in particular those caused by protein aging and/or modifications such as oxidation, deamidation, truncation, glycation, and methylation,^5^^,^^34^ may therefore be a more promising approach to explain the observed increase in T1 signal intensity of the adult lens. However, further studies are needed to investigate a potential relationship between T1 signal intensity and age-associated protein changes of the lens.

In patients with diabetes mellitus, the lens seems to increase in thickness and become more convex with age as compared with healthy subjects.^35^^,^^36^ Therefore patients with a history of diabetes were excluded from our study to generate normative data of the aging lens. The origin of the increase in diabetic lens size remains unclear. Apart from accelerated growth of the lens, possible explanations include a decrease in the central compaction of the mature lens fibers or swelling owing to increased water content without major focal loss of transparency.^35^^,^^37^^,^^38^ The finding that diabetes mellitus is associated with an increase in lens thickness is also consistent to reports of diabetes mellitus being associated with higher rates of cataract.^39^ In addition to diabetes mellitus, hypertension is not only an important risk factor for cataract formation,^40^^,^^41^ but also may even aggravate the negative impact of diabetes mellitus on cataract progression.^42^ A study by Lee et al.^43^ reported that hypertension exacerbate cataract formation by modifying protein secondary structures in the lens capsule, thereby causing alteration of membrane transport and permeability for ions. Therefore, we added hypertension as a confounding variable in the linear regression analysis and found no significant influence of hypertension on normalized T1 signal intensity of the lens in the adult cohort. In addition to that factor, age, gender, and indication for MRI of the brain had no significant influence on the equatorial diameter in the children group and on the normalized T1 signal intensity of the crystalline lens in the adult group. However, it should be noted that other important potential confounders, such as refraction, axial length, and the presence of cataract, were not known and could therefore not be included.

Further limitations of this study have to be acknowledged. This was a retrospective study with a small sample size of 30 adults and 47 children. Further studies with a larger number of patients from multiple centers will be required to verify the findings. Another limitation of this study was the analysis of T1-weighted images acquired by two MRI scanners with different field strength (1.5 vs. 3.0 Tesla) as well as significantly different acquisition protocols (e.g., repetition times of 1120 ms vs. 2000 ms). Consequently, the absolute signal intensity values might not be comparable.^44^ We, therefore, normalized the signal intensity of the lens and vitreous body by the signal intensity of the cerebrospinal fluid, similar to an approach described by Kasper et al.,^45^ to account for potential scanner-dependent differences. Furthermore, asymmetries of the lens were not taken into account. Additional studies with image-based volumetry of the human lens could be helpful to accurately assess lens asymmetry. Moreover, in this study all measurements were performed on brain MRI scans with 0.9-mm spatial resolution instead of ocular MRI scans with normally higher spatial or in plane resolutions ranging from 0.1 to 0.3 mm as in previous MRI studies.^24^^,^^25^^,^^46^ The use of ultra-high-field MRI allows even higher spatial resolutions, such as 0.25 mm and 0.70 mm at 7 Tesla, but is mostly used for research purposes only.^7^^,^^47^ Owing to the lower spatial resolution in our study, we cannot rule out that partial volume effects may have a greater effect on the accuracy of the measurements compared with studies with higher spatial resolution. It also cannot be ruled out that patient suffered from undocumented eye disease and were incorrectly included in the study cohort. Finally, the accommodative state of the human eye during propofol anesthesia is still quite unknown and cannot definitely be determined in this study owing to its retrospective character*.*

In conclusion, our study demonstrated that advancing age was significantly correlated with increasing equatorial diameter of the infantile lens and with increasing normalized signal intensity on T1-weighted images of the adult lens, also after accounting for potential confounding variables. These normative data can contribute to our understanding of age-related changes in eye health and function, especially in regard to the emmetropization process and should also be taken into account when investigating lens pathologies.

## Supplementary Material

Supplement 1
