# Supplementary material for: Age-Related Changes of the Human Crystalline Lens on High-Spatial Resolution Three-Dimensional T1-Weighted Brain Magnetic Resonance Images In Vivo
Source: Invest Ophthalmol Vis Sci. 2020 Dec 3;61(14):7. doi: 10.1167/iovs.61.14.7 (PMC7718815; doi:10.1167/iovs.61.14.7)
Supplement: Supplement 1 [file iovs-61-14-7_s001.pdf]

Supplementary Table 1: MR acquisition parameters.

Parameters	1.5 Tesla	3 Tesla
**Repetition time (ms)**	1120	2000
**Echo time (ms)**	3	2.32
**Field of view (mm)**	250 x 250	240 x 240
**Matrix size**	256 x 256	256 x 256
**Slice thickness (mm)**	0.9	0.9
**Acquisition time (min)**	03:35	04:37
**Flip angle (°)**	15	8
**Inversion time (ms)**	600	900
